# The Mitochondrial Complexome of *Medicago truncatula*

**DOI:** 10.3389/fpls.2013.00084

**Published:** 2013-04-15

**Authors:** Leonard Muriithi Kiirika, Christof Behrens, Hans-Peter Braun, Frank Colditz

**Affiliations:** ^1^Department of Plant Molecular Biology, Institute for Plant Genetics, Leibniz University HannoverHannover, Germany; ^2^Department of Plant Proteomics, Institute for Plant Genetics, Leibniz University HannoverHannover, Germany

**Keywords:** *Medicago truncatula*, mitochondrial complexome, 2D BN/SDS-PAGE, GelMap annotation tool, mitochondrial prohibitins

## Abstract

Legumes (Fabaceae, Leguminosae) are unique in their ability to carry out an elaborate endosymbiotic nitrogen fixation process with rhizobia proteobacteria. The symbiotic nitrogen fixation enables the host plants to grow almost independently of any other nitrogen source. Establishment of symbiosis requires adaptations of the host cellular metabolism, here foremost of the energy metabolism mainly taking place in mitochondria. Since the early 1990s, the galegoid legume *Medicago truncatula* Gaertn. is a well-established model for studying legume biology, but little is known about the protein complement of mitochondria from this species. An initial characterization of the mitochondrial proteome of *M. truncatula* (Jemalong A17) was published recently. In the frame of this study, mitochondrial protein complexes were characterized using Two-dimensional (2D) Blue native (BN)/SDS-PAGE. From 139 detected spots, the “first hit” (=most abundant) proteins of 59 spots were identified by mass spectrometry. Here, we present a comprehensive analysis of the mitochondrial “complexome” (the “*protein complex proteome*”) of *M. truncatula via* 2D BN/SDS-PAGE in combination with highly sensitive MS protein identification. In total, 1,485 proteins were identified within 158 gel spots, representing 467 unique proteins. Data evaluation by the novel GelMap annotation tool allowed recognition of protein complexes of low abundance. Overall, at least 36 mitochondrial protein complexes were found. To our knowledge several of these complexes were described for the first time in *Medicago*. The data set is accessible under http://www.gelmap.de/medicago/. The mitochondrial protein complex proteomes of *Arabidopsis* available at http://www.gelmap.de/arabidopsis/ and *Medicago* are compared.

## Introduction

Mitochondria are of great importance for ATP production in eukaryotic cells. Redox equivalents in the form of NADH and FADH are re-oxidized by the mitochondrial respiratory chain located in the inner mitochondrial membrane. These reactions are conducted particularly by large protein complexes forming the Oxidative Phosphorylation (OXPHOS) system, which transfer electrons to molecular oxygen. Coevally, a proton gradient is generated across the membrane. The backflow of protons into the mitochondrial matrix space mediates phosphorylation of ADP by the ATP synthase complex. A special feature of plant mitochondria is the presence of additional “alternative” oxidoreductases in the OXPHOS system (Heazlewood et al., 2003a; Brugière et al., 2004). Besides OXPHOS, mitochondria carry out additional biochemical functions, like amino acid and nucleotide metabolism, as well as synthesis of cofactors such as heme, biotin, lipoic acid (Dubinin et al., 2011). In plants, mitochondria also carry out some reactions of the photorespiratory pathway (glycolate cycle). The protein complement of *Arabidopsis*, potato, rice, and pea mitochondria have been analyzed extensively by gel-based and gel-free proteomic approaches (Klodmann et al., 2011). Many of the enzymes present in mitochondria are organized in the form of protein complexes.

Two-dimensional (2D) Blue native (BN)/SDS-PAGE is an excellent system for the separation of mitochondrial protein complexes in their native forms and subsequent resolution into their subunits (Klodmann et al., 2011). Using this approach, individual protein complexes of the respiratory chain of plant mitochondria were systematically characterized [e.g., characterization of complex I (Heazlewood et al., 2003b; Meyer et al., 2008; Klodmann et al., 2010; Klodmann and Braun, 2011); characterization of protein complex abundances of complexes I to V in different organs of *Arabidopsis* (Peters et al., 2012)]. By combining 2D BN/SDS-PAGE with sensitive mass spectrometry-based protein identification and subsequent annotation with the novel “*GelMap*” software tool (Senkler and Braun, 2012)^1^, a systematic characterization of protein complexes became possible. GelMap allows annotation of proteins according to functional categories, as well as assignment of entire sets of proteins to individual protein spots (Klodmann et al., 2011). GelMap was initially developed to functionally annotate proteins from 2D Isoelectric focusing (IEF)/SDS gels (Rode et al., 2011). For annotation of proteins separated *via* 2D BN/SDS-PAGE, the software was modified (Senkler and Braun, 2012) to allow visualization of protein complexes and their subunits even when they are of low abundance and/or are covered by higher abundant proteins. Thus, GelMap allows the systematic stock take of the mitochondrial *protein complex proteome*, the *complexome*. In *Arabidopsis*, this led to the identification of 471 distinct mitochondrial proteins and more than 35 different protein complexes (Klodmann et al., 2011).

Legumes frequently interact with soil-borne microbes (Colditz and Braun, 2010). Foremost the legume rhizobia (LR) symbiosis is of high economic value, since LR provides the host legume with independence of other nitrogen sources and helps in the production of protein-rich fruits and seeds. However, it strongly relies on the energy metabolism of the host cells (Dubinin et al., 2011). Since most of the microbial interactions to legumes are located in the rhizosphere, particularly the hosts’ root cells are in the focus of molecular research. Differences in the protein patterns of root-derived cell suspension cultures from the model legume *M. truncatula* were observed after inoculation with spores from an oomycete pathogen (Trapphoff et al., 2009). They closely match those of infected plant root cells. Thus, these root-derived cell suspension cultures may be used as an adequate model system for microbe – plant interaction studies. To date, only few studies investigated cellular sub-proteomes from the legume plant family. For example, the response of the pea mitochondrial proteome to abiotic stress conditions was investigated (Taylor et al., 2005). More recently, root plastids from *M. truncatula* were proteomically analyzed (Daher et al., 2010). The first proteomic reference maps (*via* 2D IEF/SDS-PAGE and BN/SDS-PAGE) for purified mitochondrial fractions were established by Dubinin et al. (2011). This study used the “first hit” (=most abundant) proteins from MALDI-TOF MS/MS for each analyzed protein spot.

Recently, the draft sequence of the *M. truncatula* euchromatin was published, covering almost 95% of all predicted genes (Young et al., 2011). A new database for *Medicago* DNA sequences, LegProt db, was established (Lei et al., 2011). As a consequence, chances of identifying proteins based on MS analyses of tryptic peptide mixtures of *Medicago* samples improved considerably and this database was also used for protein identification presented in this study. At the same time, sensitivity of MS systems used for protein analyses increased. Finally, the GelMap software tool for the first time allows extensive annotation of gel-based proteome data. Together, these developments sparked a carefully re-analysis of the mitochondrial proteome of *Medicago*.

## Materials and Methods

### Preparation of mitochondria from *M. truncatula*, 2D BN/SDS-PAGE

Mitochondria were isolated from *M. truncatula* (“Jemalong A17”) root cell suspension cultures as described by Dubinin et al. (2011). For 2D BN/SDS-PAGE, aliquots of isolated mitochondria equivalent to 1 mg protein were used. BN electrophoretic separation of mitochondrial protein complexes was performed according to Schägger and von Jagow (1991) with modifications (Dubinin et al., 2011) using a Protean II (16 cm × 16 cm) electrophoresis chamber (BioRad), a polyacrylamide concentration gradient of 4.5–16% acrylamide (top to bottom) for BN first dimension gel and 16.5% acrylamide Tricine/SDS-PAGE for the second gel dimension. Gels were stained with Coomassie blue-colloidal (BioRad) overnight and scanned on an UMAX Power Look III Scanner (UMAX Technologies) as described before (Colditz et al., 2007).

### Mass spectrometry

Protein spots of 1.4 mm diameter were cut from Coomassie-stained gels using a GelPal Protein Excision manual spot picker (Genetix, Great Britain) and in-gel digested with Trypsin as described by Klodmann et al. (2010). Tryptic peptides were further analyzed by nanoHPLC (Proxeon, Thermo Scientific) coupled to electrospray ionization quadrupole time of flight MS (micrOQTOF Q II, Bruker Daltonics), using all settings and parameters as described previously (Klodmann et al., 2011). Data processing and protein identification was carried out with ProteinScape 2.0 (Bruker Daltonics) and the MASCOT search engine querying three *Medicago*-specific protein databases [*Mt3.5 ProteinSeq, NCBI Medicago truncatula protein, and Mtf*(*asta*)^2^] available at the LegProt db (Lei et al., 2011) as well as *Swiss Prot*, using the following parameters: trypsin/P; one missed cleavage allowed; fixed modifications: carbamidomethylation (C), variable modifications: acetylation (N) and oxidation (M); precursor ion mass tolerance, 30 ppm; peptide score >24; charges 1+, 2+, 3+. Protein and peptide assessments with MASCOT scores above 25 were considered. Identified proteins were further analyzed for their sub-cellular localization using their homologous *Arabidopsis* accessions (according to TAIR 10 db) queried against the SUBA III database (Heazlewood et al., 2007)^3^.

### Mitochondrial BN reference map *via* GelMap

After protein identification, the reference map was visualized using the GelMap platform (see text footnote 2; Senkler and Braun, 2012). For this purpose, spots of a scanned 2D BN/SDS gel were automatically detected and were given consecutive spot numbers with corresponding *x*- and *y*-coordinates by the Delta 2D (4.2) software (Decodon, Greifswald, Germany) (Figures S1–S3 in Supplementary Material). An Excel (Microsoft) file containing this information and the corresponding gel image (.jpg) were then imported into GelMap. MS/MS results were uploaded as well. Detailed information on building a GelMap is available under http://www.gelmap.de/howto.

## Results and Discussion

### 2D BN/SDS-PAGE of mitochondrial protein fractions from *M. truncatula* cells

**Figure 1:**
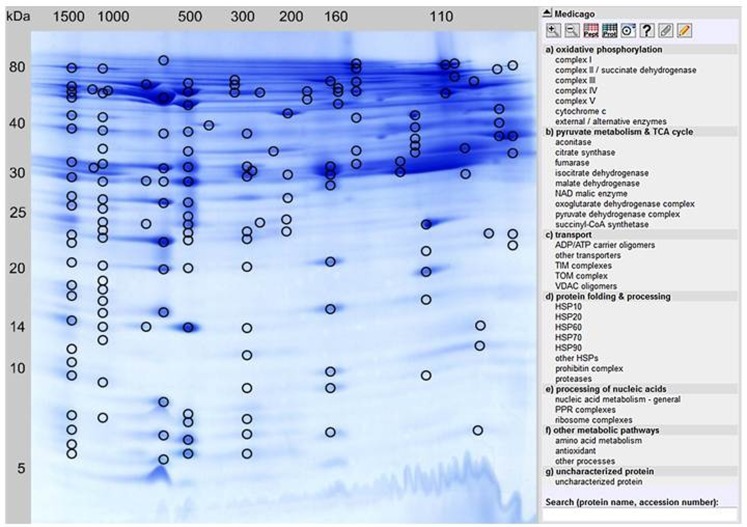
**GelMap reference map of the *M. truncatula* mitochondrial protein complexe proteome/complexome (http://www.gelmap.de/medicago/)**. Hundred and fifty-eight protein spots separated by 2D BN/SDS-PAGE and identified by MS are marked by circles. Most protein spots include multiple protein annotations. By clicking a certain protein spot, all identified proteins within this spot are shown in a pop-up window, beginning with the protein identification of the highest MASCOT score. The menu to the right lists classes of physiological functions for mitochondrial protein complexes. By clicking on the selected protein complex in this menu, accessions of all included individual proteins as well as the corresponding protein spots in the gel image are highlighted. Alternatively, a protein can be found in GelMap by the Search tool at the bottom to the right.

In order to separate mitochondrial proteins from *M. truncatula* root-derived cell suspension cultures, purified mitochondrial fractions were prepared according to an optimized protocol published by Dubinin et al. (2011). Proteins from five independent mitochondrial isolations were then separated by 2D BN/SDS gel electrophoresis. Spot patterns on the gels were highly similar as revealed by Delta 2D analysis (data not shown). From this set of five, a representative gel was selected for MS analyses as well as for online data presentation *via* the GelMap software tool (Figure 1)^4^. Using the same gel, a spot coordinate file was generated as described in Section “Materials and Methods” and uploaded simultaneously.

### MS-based protein identification and annotation of *M. truncatula* mitochondrial proteins

All 158 protein spots encircled in Figure 1 were analyzed *via* nLC ESI-MS measurements. In contrast to the previous analysis of the 2D BN/SDS-PAGE-separated mitochondrial proteome by Dubinin et al. (2011), protein identification was achieved using the *Medicago*-specific protein databases from the LegProt db (Lei et al., 2011), resulting in improved protein identification rates. In total, 1,485 proteins were identified within the selected protein spots, representing 467 unique proteins. For nine proteins, no accessions were found in MtGI. Interestingly, 12 of the uniquely identified proteins in MtGI have no homologs in *Arabidopsis* (spots 47, 75, 76, 77, 85, 98, 101, 106, 113, 116, 132, 133, 137). Among them are three legume-specific proteins involved in symbiosis to Rhizobial bacteria: a legume lectin (ID 101) and two nodulins (nodulin 3, spot 106; nodulin 25, spot 85), as well as a prefoldin protein (spot 77) which is supposed to be also legume-specific. The majority of plant lectins possess a signal peptide and thus are targeted *via* the secretory pathway into the vacuolar and extracellular compartments (Lannoo and Van Damme, 2010). Recently, evidence was given that plants additionally synthesize small amounts of lectins in response to changing environmental conditions or stress factors, which are referred to as “inducible” lectins (Lannoo and Van Damme, 2010). Contrary to the majority of plant lectins, these inducible lectins have been shown to be located to the cytosolic/nuclear compartment, and even their involvement in mitochondrial-induced programed cell death (PCD) has been reported (Van Damme et al., 2004). How these proteins are involved in mitochondrial metabolism should be analyzed in future studies. Nevertheless, we cannot exclude that the identified lectins are contaminants in our mitochondrial fractions. In addition, two *Medicago* hexokinases (hexokinase 7, spots 98a and 113; hexokinase 8, spots 113 and 132) have no homologoues in *Arabidopsis*.

By clicking a spot in Figure 1, the description(s) of the identified protein(s) will appear in a pop-up window. These descriptions are hyperlinked and by pointing the mouse cursor at any one of them a detailed information is provided. This includes: spot number, protein name, MS score, calculated and apparent molecular mass (for both gel dimensions), sequence coverage, number of matching peptides, the Tentative Consensus (TC) accession from the *Medicago truncatula Gene Index* [MtGI, Release 11.0 (March 23, 2011), at Dana-Farber Cancer Institute, Harvard School of Public Health, Boston, MA, USA], the TAIR accession from the *Arabidopsis* homologous protein, protein name and origin, the protein database where the protein was identified, its protein complex identity, physiological function, and sub-cellular localization according to the SUBA III database (Heazlewood et al., 2007). Most protein spots shown in Figure 1 contain several different proteins. Within the pop-up window of each spot, they are sorted according to their relative abundances, as implicated by their respective Mascot scores. Most abundant proteins are listed at the top, least abundant proteins at the bottom. By showing all identified protein hits GelMap promotes the detection of low abundant protein complexes which cannot be found when only the most abundant hits per spot are considered. In case of multiple protein annotation for an individual spot, another mouse-click on the protein of choice opens a new window that includes detailed information. For several proteins, identification in MtGI was not yet possible. In these cases, heterologous protein identifications of the most homologous *Arabidopsis* proteins/accessions are given.

In order to assess the purity of isolated mitochondrial fractions, the sub-cellular localization of all proteins identified was evaluated *via* the Sub-Cellular Proteomic Database (SUBA III, see text footnote 4). Since this database collects experimental data and in silico predictions of the localization of proteins in *Arabidopsis*, the corresponding TAIR homologs of each identified *Medicago* protein were used to assess the intracellular whereabouts of the *Medicago* proteins. At least for the “first hit” identifications, prediction data are available (except for one of the 158 “first hit” proteins). From overall 157 first protein hits, 145 proteins (92%) are assigned to mitochondria. Considering all 467 unique proteins, sub-cellular localization information is available for 413 proteins. The percentage of mitochondrial proteins in this dataset is lower (287 proteins = 69.5%). Twenty-five proteins (=6%) represent cytosolic proteins according to SUBA evaluation. A considerable number of proteins are assigned to other cellular compartments: 6% to plastids, 5% to the nucleus, 3.6% to membrane structures (plasma membrane, endomembrane), and 1.2% to the cells vacuoles. For 17 proteins (4%), no SUBA predictions are available because of a lack of experimental data. These proteins are labeled as “NEW mitochondria” in our GelMap since they represent candidates for mitochondrial proteins. Considering that the “first hit” proteins, 92% of which are of predicted mitochondrial origin, are on average significantly more abundant than the proteins of lower MASCOT scores within each spot, we estimate that the overall purity of our mitochondrial fraction was in the range of 85%.

### Annotation of the *M. truncatula* mitochondrial complex proteome/complexome

Two-dimensional BN/SDS reference maps generated with GelMap enable annotation and assignment of all proteins identified that belong to one certain functional protein complex, for example the complexes of the OXPHOS system (Klodmann et al., 2011). Systematic evaluation of all apparent protein complexes allows establishing the complexome of the protein sample.

For this purpose, the “physiological function” menu to the right of the GelMap (Figure 1) should be used. Here, functional classification of all identified subunits is given, next to their assignment to protein complexes. According to our GelMap evaluation, 36 mitochondrial protein complexes were found in the *Medicago* mitochondrial fractions. Several of these protein complexes were described for the first time in this model legume.

### Evaluation of the *Medicago* mitochondrial protein complex proteome *via* GelMap

The GelMap of the *M. truncatula* mitochondrial complex proteome presented here aims to systematically analyze the complexome of this sub-cellular compartment. Since the GelMap annotation portal is web-based, the data set is open to the scientific community and public data evaluation is possible and welcome. The *Medicago* mitochondrial GelMap includes proteins with MASCOT scores ≥25 as well as proteins identified by one single peptide in order to provide a maximum of information. Thus, the currently presented data should be treated with caution because false positive identifications are not completely excluded. At the same time, for some proteins, MS-spectra were recorded but no positive identification was possible from the data. To overcome both of these drawbacks we will continuously update this protein reference map when progress in the annotation of *Medicago* genome allows better identification of proteins.

While *Medicago* is still trailing *Arabidopsis* in respect to genome annotations, the data produced in this study nevertheless allow a comparison of the mitochondrial complex proteome of both species. Most complexes found in *Arabidopsis* are also present in *Medicago*, which is not surprising given the importance of mitochondrial function for the energy metabolism of plants. However, some protein complexes of *Medicago* mitochondria seem to lack comparable counterparts in *Arabidopsis*.

**Figure 2:**
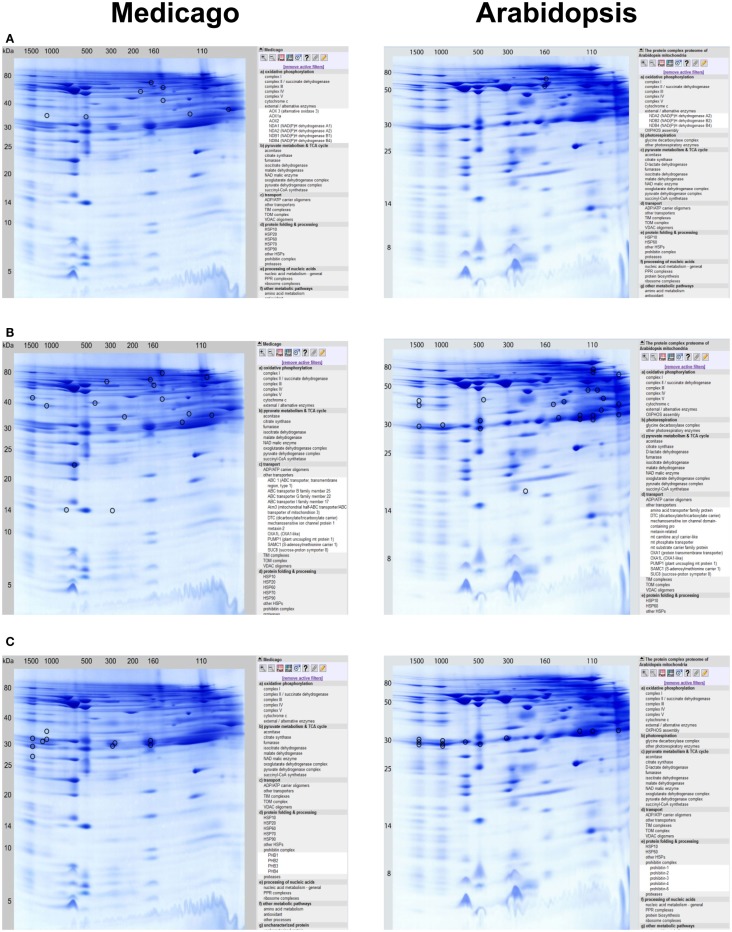
**Detailed protein complex visualization in the GelMap reference maps of the *Medicago* mitochondrial complexome (http://www.gelmap.de/medicago/, left row) as compared to the *Arabidopsis* mitochondrial complexome (http://www.gelmap.de/arabidopsis/, right row)**. Visualization of protein complexes and all of its included individual proteins is exemplarily done for: **(A)** external/alternative enzymes, **(B)** other transporters/ABC transporters, and for **(C)** prohibitin complexes.

A short overview and characterization of the major *Medicago* protein complexes is given below:

– *Complex I* is the biggest respiratory chain complex. It runs at 1000 kDa and 41 of its subunits could be identified, although some subunits seem to be missing since more were found in *Arabidopsis* (Klodmann et al., 2010). Several of its subunits are plant specific, e.g., the gamma carbonic anhydrases (Klodmann et al., 2010). Subcomplexes of complex I are found at MWs of approximately 500, 280, and 140 kDa, which probably represent assembly intermediates of the complex. l-galactono-1,4-lactone dehydrogenase (GLDH), which was recently described to form part of three assembly intermediates of complex I in *Arabidopsis* (Schertl et al., 2012), also does not form part of fully assembled complex I in *Medicago*.
– Complex I interacts with dimeric cytochrome c reductase to form the *I + III_2_* *supercomplex* of 1500 kDa.
– *Complex II* is resolved in its main form at 160 kDa, but an additional version is present at 110 kDa. The occurrence of this smaller form of complex II was described before by Dubinin et al. (2011) for *Medicago*, and by Klodmann et al. (2011) for *Arabidopsis*. Overall, five different complex II subunits were identified, including the two plant specific subunits SDH5 and SDH7-2. According to Dubinin et al. (2011), the relative abundance of the two forms of complex II differs between *Medicago* and *Arabidopsis*, which was confirmed by our new study.
– *Complex III* is a dimer in its active form and runs at 500 kDa. Nine of the expected 10 subunits were found. With complex I, it is involved in the formation of a supercomplex.
– Of *complex IV*, six different subunits were identified.
– *Complex V* (ATP synthase) can be found at 600 kDa on the 2D BN/SDS gel. Thirteen distinct subunits were identified. As described previously by Dubinin et al. (2011), the complex V dimer (V_2_) is of higher abundance in mitochondria isolated from *Medicago* cells than in *Arabidopsis*.
– Several of the *alternative oxidoreductases of the plant OXPHOS system* were identified: AOX1a, AOX2, AOX3, NDA1, NDA2, NDB1, and NDB4. The NDA and NDB subunits are present at 160kDa and probably form a protein complex as reported for *Arabidopsis* (Klodmann et al., 2011). AOX is found at many different horizontal positions in the *Medicago* GelMap, possibly indicating its binding to a variety of protein complexes (Figure 2A).
– Since *cytochrome c* migrates at low MW (15 kDa) in the second gel dimension, but at much higher MWs in the native first dimension (100–200 kDa), indication for an association with other proteins (complex IV subunits, GLDH) is given.
– Besides the membrane-bound protein complexes, several enzymes of the *citric acid cycle* also are involved in forming protein complexes: aconitase forms a putative dimer at 150 kDa in the native gel dimension, NAD-dependent malic enzymes was described to form a heterohexamer in *Arabidopsis* at 369 kDa (Klodmann et al., 2011), which was also found in *Medicago*. A pyruvate dehydrogenase E1 and E3 subcomplex was detected at 140 kDa, which has also been described in *Arabidopsis* (Klodmann et al., 2011).
– Several *ADP/ATP carrier* oligomers were identified at MWs between 90 and 110 kDa on the native gel dimension.
– Interestingly, several *ABC transporters* identified in the MW range between 160 and 780 kDa were found, which were not described in the *Arabidopsis* mitochondrial proteome (Figure 2B).
– *TIM* and *TOM* protein complexes: the TOM complex containing the subunits TOM20-2, TOM20-3, and TOM22-V was found at a MW of 260 kDa. In addition, TOM complex subunits (TOM20-2, TOM40) were found at MWs of 1000 and 1500 kDa. In the same MW range, also TIM subunits were found (TIM17-22, TIM17-2), suggesting the presence of a large TIM/TOM translocon supercomplex in *Medicago*.
– Several *VDAC* oligomers were identified between 90 and 500 kDa, which form either distinct complexes or artificially aggregated during solubilization or electrophoresis of the native gel dimension.
– *HSP*s form protein complexes in *M. truncatula* mitochondria: HSP60 complex is present in its main form at a MW of 600 kDa, where also other HSPs (HSP70, HSP90) were detected.
– Three distinct *prohibitin complexes* were found at 160, 300, and 1200 kDa. Increased abundance of mitochondrial prohibitins in *M. truncatula* was reported previously (Dubinin et al., 2011); presence of varying prohibitin complexes was not found in *Arabidposis* (Figure 2C). Inoculation of *Medicago* cells with virulent spores of an oomycete pathogen (according to Trapphoff et al., 2009) resulted in accumulation and increased abundance of the prohibitin protein complex in BN gels (F. Colditz, unpublished). Recent findings indicate that prohibitins are involved in mediating stress tolerance (abiotic stress, pathogen infection, and elicitor signaling) as well as triggering retrograde signals in response to mitochondrial dysfunction (Van Aken et al., 2010).
– *Proteases*: AAA-type ATPase family protein was found at 1500 kDa, together with two cysteine proteinases. ClpA/ClpB protease subunits are present between 150 and 600 kDa, the LON protease at 600 kDa.
– Interestingly, nine different *PPR proteins* involved in nucleic acid metabolism were identified at molecular masses ranging from 90 to 1500 kDa. They form part of so far unknown protein complexes.
– Eight distinct subunits of *ribosomal protein subcomplexes* were identified between 50 and 1000 kDa.
– A large set of *antioxidant proteins* like SOD, glutathione S-transferase and catalase were found in the molecular mass range of 120–1500 kDa on the native gel dimension, indicating their presence within protein complexes.
– Many further enzymes listed under “further metabolic pathways” much likely form part of *other protein complexes*, since they migrate at significantly higher molecular masses on the native (BN) gel dimension than on the denaturing gel dimension.

## Conclusion

This GelMap was built to systematically define the mitochondrial protein complex proteome of the model legume *M. truncatula*. Generally, our GelMap presents protein candidates that may form protein complexes. It does not provide final proof for the presence of novel complexes: if a protein complex is described here for the first time, its occurrence should be verified by further independent experiments.

Most of the identified protein complexes to be present in *Medicago* mitochondria were already identified and characterized in mitochondria from *Arabidopsis* cell suspension cultures (Klodmann et al., 2011). However, some protein complexes found in *Medicago* mitochondria, such as ABC transporters, TIM/TOM translocon supercomplex, and distinct prohibitin complexes, seem to lack comparable counterparts in *Arabidopsis*.

Since molecular studies with legumes are particularly done to characterize interactions of plants to soil-borne microbes (Colditz and Braun, 2010), the *Medicago* mitochondrial GelMap should promote the analysis of infection-related proteomic alterations at a sub-cellular level. As a next step, analyses of the *Medicago* mitochondrial proteome after microbial infections should be carried out, especially after infection with agronomically important and legume-specific rhizobial bacteria, in order to monitor adaptive changes in the protein complement of this sub-cellular compartment.

## Conflict of Interest Statement

The authors declare that the research was conducted in the absence of any commercial or financial relationships that could be construed as a potential conflict of interest.

## Supplementary Material

The Supplementary Material for this article can be found online at: http://www.frontiersin.org/Plant_Proteomics/10.3389/fpls.2013.00084/abstract

Supplementary Figure S1 **Molecular mass scale for the 2D gel used for calibration and generation of *M. truncatula* mitochondria GelMap**.

Supplementary Figure S2 **Mitochondria protein complexes of *M. truncatula* resolved by 2D blue native/SDS PAGE**.

Supplementary Figure S3 **Spot detection on the 2D BN/SDS gel done automatically using DELTA 2D software package (version 4.3.2)**.

## Footnotes

^1^http://www.gelmap.de/

^2^http://bioinfo.noble.org/manuscript-support/legumedb/

^3^http://suba.plantenergy.uwa.edu.au/

^4^http://www.gelmap.de/medicago/
