# Supplementary material for: The Mitochondrial Complexome of Medicago truncatula
Source: Front Plant Sci. 2013 Apr 15;4:84. doi: 10.3389/fpls.2013.00084 (PMC3625726; doi:10.3389/fpls.2013.00084)

The mitochondrial complexome of *Medicago truncatula*

Leonard Muriithi Kiirika[1], Christof Behrens[2], Hans-Peter Braun[2] and Frank Colditz[1]*
[1] *Department of Plant Molecular Biology, Institute for Plant Genetics, Leibniz University Hannover, Hannover, Germany*
[2] *Department of Plant Proteomics, Institute for Plant Genetics, Leibniz University Hannover, Hannover, Germany*

Supplementary material

Supplementary figures:

Figure S1.

Molecular mass scale for the 2D gel used for calibration and generation of *M. truncatula* mitochondria GelMap

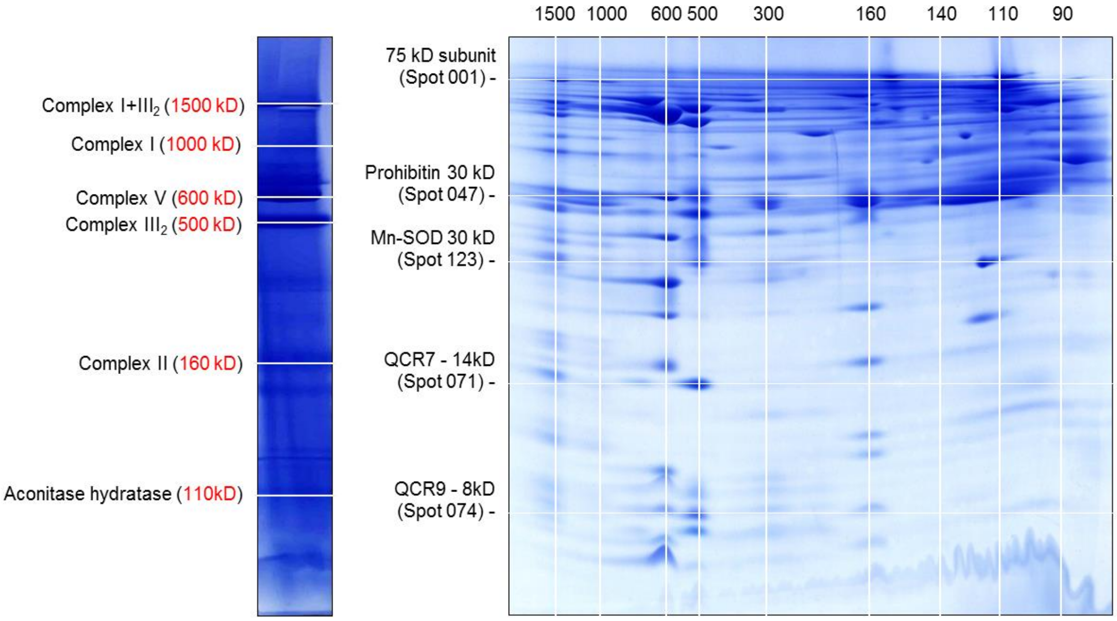

a) Molecular mass scale for BN gel dimension

b) Molecular mass scale for 2D gel dimension

Figure S2.

Mitochondria protein complexes of *M. truncatula* resolved by 2D blue native / SDS PAGE

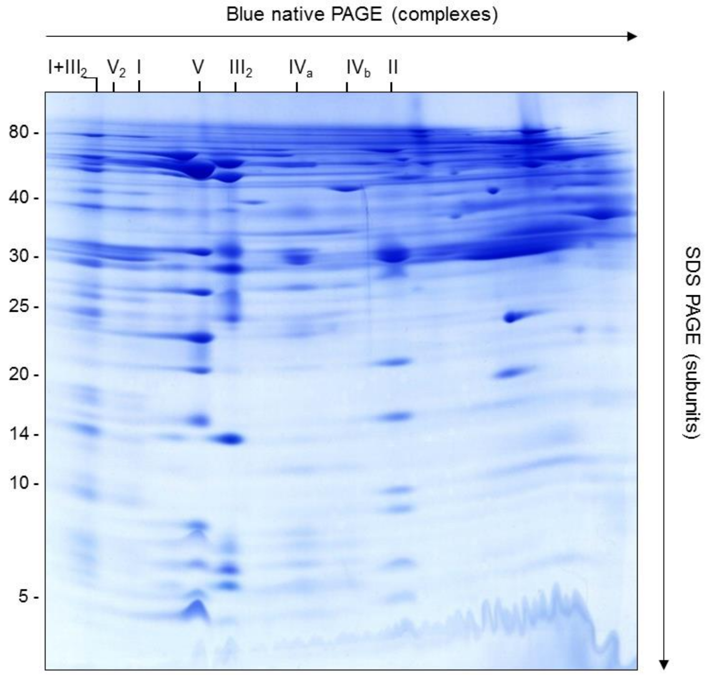

Figure S3

Spot detection on the 2D BN / SDS gel done automatically using DELTA 2D software package (version 4.3.2).

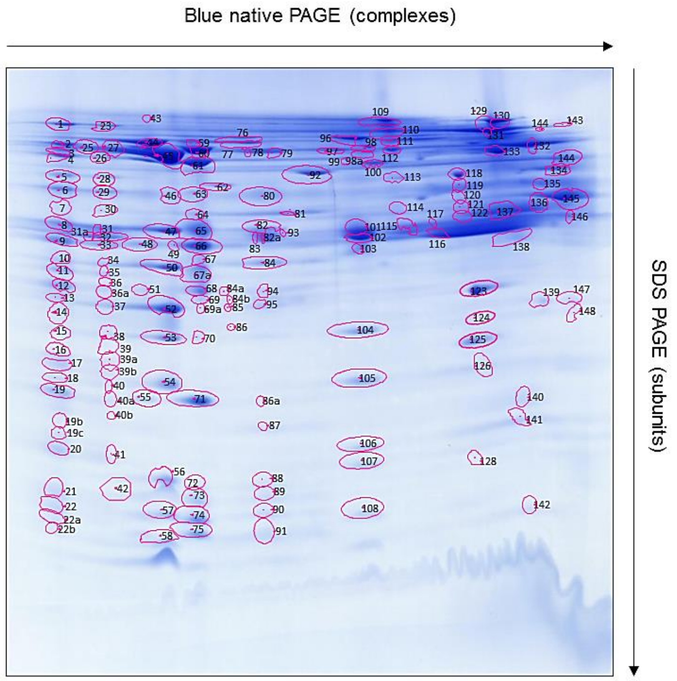

Supplement: Supplementary Figure S1 — Molecular mass scale for the 2D gel used for calibration and generation of M. truncatula mitochondria GelMap. [file 45988_Colditz_Presentation1.PDF]
